# ClpC affects the intracellular survival capacity of *Staphylococcus aureus* in non-professional phagocytic cells

**DOI:** 10.1038/s41598-019-52731-3

**Published:** 2019-11-07

**Authors:** Gubesh Gunaratnam, Lorena Tuchscherr, Mohamed I. Elhawy, Ralph Bertram, Janina Eisenbeis, Christian Spengler, Thomas Tschernig, Bettina Löffler, Greg A. Somerville, Karin Jacobs, Mathias Herrmann, Markus Bischoff

**Affiliations:** 1 Institute of Medical Microbiology and Hygiene, Saarland University, Homburg/Saar, Germany; 2 Institute of Medical Microbiology, Jena University Hospital, Jena, Germany; 3 Institute of Clinical Hygiene, Medical Microbiology and Infectiology, Paracelsus Medical University, Nuremberg, Germany; 4 Experimental Physics, Saarland University, Saarbrucken, Germany; 5 Institute of Anatomy and Cell Biology, Saarland University, Homburg/Saar, Germany; 6 School of Veterinary Medicine and Biomedical Sciences, University of Nebraska-Lincoln, Lincoln, Nebraska USA; 7 Institute of Medical Microbiology, University Hospital of Münster, Münster, Germany

**Keywords:** Infection, Bacterial immune evasion

## Abstract

Invasion and persistence of bacteria within host cells requires that they adapt to life in an intracellular environment. This adaptation induces bacterial stress through events such as phagocytosis and enhanced nutrient-restriction. During stress, bacteria synthesize a family of proteins known as heat shock proteins (HSPs) to facilitate adaptation and survival. Previously, we determined the *Staphylococcus aureus* HSP ClpC temporally alters bacterial metabolism and persistence. This led us to hypothesize that ClpC might alter intracellular survival. Inactivation of *clpC* in *S. aureus* strain DSM20231 significantly enhanced long-term intracellular survival in human epithelial (HaCaT) and endothelial (EA.hy926) cell lines, without markedly affecting adhesion or invasion. This phenotype was similar across a genetically diverse collection of *S. aureus* isolates, and was influenced by the toxin/antitoxin encoding locus *mazEF*. Importantly, MazEF alters mRNA synthesis and/or stability of *S. aureus* virulence determinants, indicating ClpC may act through the mRNA modulatory activity of MazEF. Transcriptional analyses of total RNAs isolated from intracellular DSM20231 and isogenic *clpC* mutant cells identified alterations in transcription of α-toxin (*hla*), protein A (*spa*), and *RNAIII*, consistent with the hypothesis that ClpC negatively affects the intracellular survival of *S. aureus* in non-professional phagocytic cells, via modulation of MazEF and Agr.

## Introduction

The gram-positive bacterium *Staphylococcus aureus* is a common cause of persistent and relapsing infections^1^. These types of infections are hypothesized to be due to the formation of persister cells having reduced metabolic activity and virulence factor synthesis^2^. In addition, persistent and relapsing infections are thought to be aided by *S. aureus* ability to invade and survive in non-professional phagocytic cells^3,4^. Persistent *S. aureus* infections often involve the formation of small colony variants (SCV)s that have defects in electron transport and thymidylate biosynthesis^5^, a process requiring the alternative sigma factor σ^B^ ^6^. During the study of *S. aureus thyA* mutants displaying an SCV phenotype, it was observed that transcription of the heat shock protein (HSP) ClpC, a ClpATPase encoded by *clpC*, was significantly reduced relative to the isogenic parental strain^7^. This observation raised the possibility that metabolic signals created by inactivating *thyA* are transduced into action through repression of ClpC activity.

While the function of the HSP ClpC on SCV formation is unclear, its role as an effector of staphylococcal energy metabolism, stress adaptation, and stationary phase survival is established^8–10^. These effects are mediated over a broad range of genes in the *S. aureus* transcriptome^9,11^, in concert with ClpC’s cognate protease ClpP and the proteolytic adaptor protein TrfA^12^. In addition to affecting transcription, ClpC alters the activity of toxin/antitoxin systems in *S. aureus* by degrading antitoxins^12,13^, including the toxin/antitoxin system MazEF. MazF represents a sequence specific RNase, while MazE is a cognate inhibitor of MazF and subject to proteolysis to activate MazF^14^. Interestingly, the MazEF toxin/antitoxin system affects intracellular survival of *S. aureus* in osteoblasts^15^. Taken together, the effect of ClpC on metabolism, survival, and MazEF, suggests that this HSP might affect intracellular persistence. This suggestion is supported by the observation that deletion of *clpC* in *S. aureus* strain 8325-4 decreased intracellular replication in bovine mammary epithelial cells^16^; of note, this study did not address *S. aureus* persistence. To assess the significance of ClpC in *S. aureus* intracellular persistence, bacterial long-term survival in keratinocytes and endothelial cells was examined.

## Results and Discussion

### *S. aureus* strain DSM20231 adheres to and is internalized by non-professional phagocytic cells

**Figure 1:**
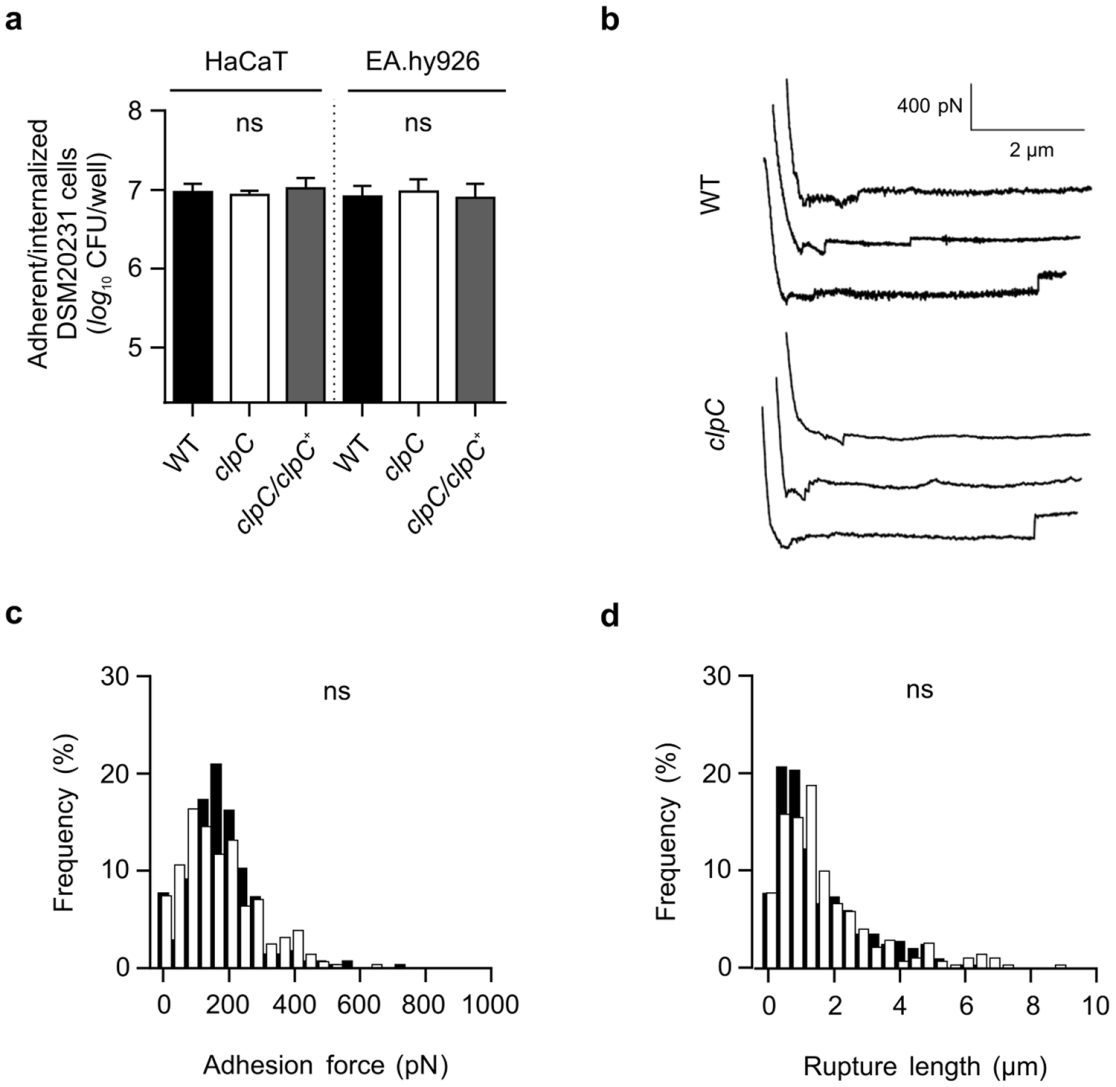
Adhesion capacities of *S. aureus* DSM20231 and its *clpC* derivatives to non-professional phagocytes. (**a)** HaCaT and Ea.hy926 cells were challenged with the *S. aureus* strains DSM20231 (WT; black bars), the *clpC* mutant PBM001 (*clpC*; white bars), and the *clpC* complemented PBM001 derivative (*clpC*/*clpC*^+^, grey bars) at a MoI of 100, respectively, and co-cultured for 90 min. Non-adherent bacteria were subsequently removed by washing. Eukaryotic cells were afterwards lysed by sonication, and CFU rates in lysates determined by plate counting. Data are presented as mean + SD (*n* = 6 biological replicates). ns, not significant (Kruskal Wallis test followed by Dunn’s post-hoc test). **(b–d)** Single cell force spectroscopy of DSM20231 (black bars) and PBM001 (white bars) cells on Ea.hy926 cells. **(b)** Representative retraction force profiles obtained by recording force-distance curves in DMEM between bacteria and Ea.hy926 cells spread on glass bottom petri dishes for 24 h. **(c,d)** Maximum adhesion force- and rupture length histograms. Histograms are from a total of 320 curves from 5 independent cell pairs per bacterial strain. They were obtained by determining, for each curve, the force of the strongest adhesive event (**c**) and the distance of the last rupture event (**d**). ns, not significant (Mann-Whitney *U* test).

**Figure 2:**
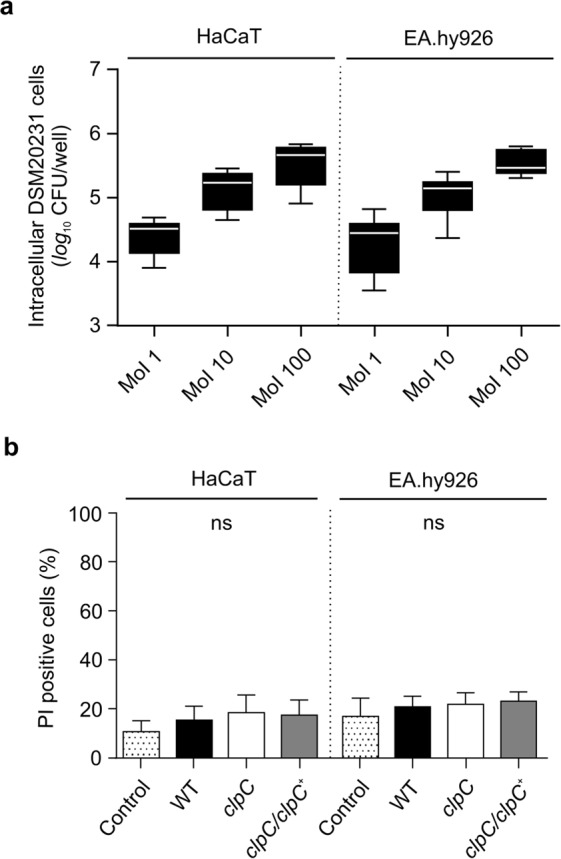
Impact of the MoI on the capacity of *S. aureus* DSM20231 to internalize non-professional phagocytes and cytotoxic potential of DSM20231 and its *clpC* derivatives on keratinocytes and endothelial cells. (**a)** Cells of the keratinocyte cell line HaCaT and the endothelial cell line Ea.hy926 were infected with *S. aureus* DSM20231 at MoIs of 1, 10, and 100, respectively, and co-cultured for 90 min. Extracellular and adherent bacteria were subsequently removed by washing and lysostaphin/gentamicin treatment, the eukaryotic cells lysed by sonication, and surviving bacteria in lysates determined by plate counting. Data are presented as box and whisker plot showing the interquartile range (25–75%; box), median (horizontal line), and whiskers (bars; min/max). (*n* = 6 biological replicates). **(b)** Eukaryotic cells were challenged with the *S. aureus* strains DSM20231 (WT; black bars), the *clpC* mutant PBM001 (*clpC*; white bars), and the *clpC* complemented PBM001 derivative (*clpC*/*clpC*^+^, grey bars) at a MoI of 100 and co-cultured for 90 min. Extracellular and adherent bacteria were subsequently removed by washing and lysostaphin/gentamicin treatment, and infected cell lines and unchallenged control cells were cultured for 24 h in cell culture medium. PI-positive cells were identified by FACS as outlined in Materials. Data are presented as mean + SD (*n* = 6 biological replicates). ns, not significant (Kruskal Wallis test followed by Dunn’s post-hoc test).

The invasion and persistence of *S. aureus* into eukaryotic cells are strongly dependent on the host cell type and the infecting *S. aureus* strain^4^. Persistence is an aggregate process that is dependent, in part, upon the bacterial abilities to adhere and be internalized by host cells. Alterations in the ability to adhere or invade would complicate the assessment of bacterial persistence. To eliminate the possibility that differences in persistence were due to changes in the competence of bacteria to adhere or be internalized, strain DSM20231 (syn. ATCC 12600), a *S. aureus* derivative of the type strain 533 R4 isolated from human pleural fluid, and known to exert a strong ClpC effect on stationary phase survival^8^, was assessed for its ability to adhere to and invade HaCaT and Ea.hy926 cells. When strain DSM20231 and its *clpC* derivatives were brought into contact with keratinocytes or endothelial cells by mild centrifugation, about one fifth of the bacteria remained associated with the eukaryotic cells after 90 min of co-incubation and washing (Fig. 1a), suggesting that bacterial adhesion to eukaryotic cells is largely independent of ClpC in *S. aureus*. To gain additional insights into the effect of ClpC on adhesion of *S. aureus* to eukaryotic cells at the single cell level, adhesion of strains DSM20231 and PBM001 to Ea.hy926 cells was also analyzed by single cell force spectroscopy (SCFS). In this method, a viable cell (here a single bacterium) is immobilized on the tip of an atomic force microscope (AFM) cantilever to create a bacterial probe. This bacterial probe is brought into physical contact with its substratum (here the extracellular matrix [ECM] of an epithelial cell), and after a certain contact time the bacterial probe is withdrawn to rupture the potentially formed bacterium-ECM interactions. The rupture force and rupture length are detected by the deflection of the AFM cantilever, which allows studying the interaction forces between a single bacterium and its substratum with nanometer spatial and piconewton force resolution^17^. Similar to the adhesion assays (Fig. 1a), the interaction forces between single bacteria and Ea.hy926 cells were comparable (Fig. 1b–d, Table S1). These data strongly suggest that *S. aureus* adhesion to eukaryotic cells is independent of ClpC and will not interfere with persistence assays. The adherence of strain DSM20231 and its mutant derivatives to HaCaT and Ea.hy926 cells was equivalent, but this does not rule out the possibility that differences in internalization could alter persistence. To address this possibility, lysostaphin/gentamicin protection assays were used. Strain DSM20231 invaded both cell lines in a bacterial cell density-dependent manner (Fig. 2a). While increasing the multiplicity of infection (MoI) enhanced bacterial invasion, invasion was not proportional to the increased number of bacteria (Fig. 2a), suggesting that the uptake process can be saturated at high MoIs. At a high MoI, bacterial cytotoxicity can kill eukaryotic cell lines. To exclude this possibility, cell viability was assessed using propidium iodide (PI) following bacterial invasion. Neither HaCaT nor EA.hy926 cells displayed increased cell death rates at 24 post infection with *S. aureus* strain DSM20231 (Fig. 2b), a result similar to that reported for *S. aureus* strains USA300, SH1000 and Cowan I^4^. Together, these results demonstrate that *S. aureus* strain DSM20231 can be reliably used in HaCaT and EA.hy926 cell lines for invasion and persistence assays.

### *S. aureus* cytotoxicity is independent of ClpC

**Figure 3:**
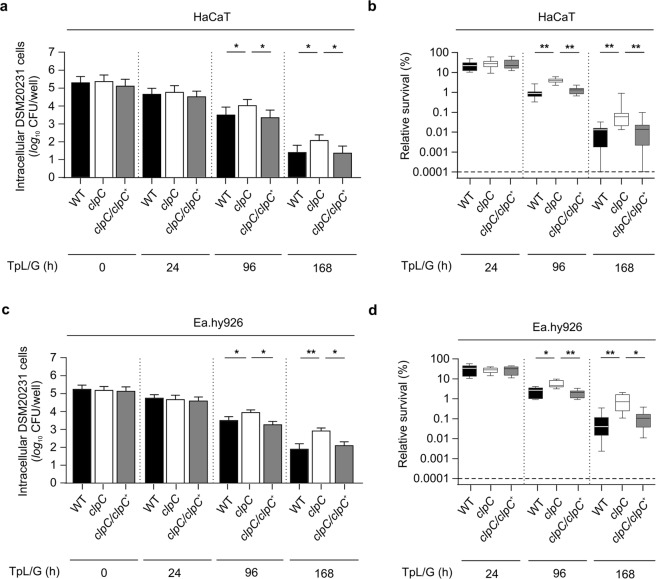
Impact of ClpC on the intracellular survival of *S. aureus* DSM20231 in keratinocytes and endothelial cells. Cells of the keratinocyte cell line HaCaT (**a, b**) and the endothelial cell line Ea.hy926 (**c, d**) were infected with the *S. aureus* strains DSM20231 (WT; black bars), the *clpC* mutant PBM001 (*clpC*; white bars), and the *clpC* complemented PBM001 derivative (*clpC*/*clpC*^+^, grey bars) at a MoI of 100, respectively, and co-cultured for 90 min. Extracellular and adherent bacteria were subsequently removed by washing and lysostaphin/gentamicin treatment, and the infected cell lines cultured for up to 168 h in cell culture medium supplemented with gentamicin. At 24, 96, and 168 h post lysostaphin/gentamicin treatment, eukaryotic cells were removed, lysed by sonication, and surviving bacteria in lysates determined by plate counting. **(a, c)** CFU rates recovered from infected HaCaT (**a**) and Ea.hy926 (**c**) lysates at the time points indicated. Data are presented as mean + SD (*n* = 8 biological replicates). **(b, d)** Relative survival (%) of intracellular bacteria in HaCaT (**b**) and Ea.hy926 (**d**) cells in relation to the CFU rates seen immediately after the lysostaphin/gentamycin treatment. Data are presented in box and whisker plots (*n* = 8 biological replicates). *P < 0.05; **P < 0.01 (Kruskal Wallis test followed by Dunn’s post-hoc test). TpL/G, time post lysostaphin/gentamicin treatment. Horizontal dashed lines indicate the limit of detection.

To determine if ClpC affects invasion of keratinocytes and endothelial cells through altered cytotoxicity, HaCaT and EA.hy926 cells were challenged with strains DSM20231, PBM001, and the *clpC* complemented PBM001 derivative, and the cells were detached and viability was assessed by propidium iodide (PI) staining. All three strains were internalized into HaCaT or EA.hy926 cells with comparable efficiencies and equivalent numbers of cells retained the PI stain (Fig. 3a, c, 0 h). To determine the cytotoxic effects of *S. aureus* infection in these eukaryotic cell lines, MTT (3-[4.5-dimethylthiazol-2-yl]-2.5-diphenyltetrazolium bromide) based cell viability assays were performed. The metabolic activities of strain DSM20231 and PBM001 infected HaCaT cultures, at 96 h and 168 h post-infection, were equivalent to those of the controls (Fig. S1), indicating that the viability of this keratinocyte cell line is not markedly affected by the intracellular persisting *S. aureus* cells. EA.hy926 cultures infected with strains DSM20231 and PBM001, however, decreased cell metabolism later during persistence (*i.e*., 96 h and 168 h post infection) relative to the bacteria-free controls (Fig. S1b). Taken together, these data indicate that ClpC is not required for internalization and that it minimally alters cytotoxicity to non-professional phagocytic cells.

### Deletion of *clpC* in *S. aureus* enhances intracellular survival in keratinocytes and endothelial cells

The absence of differences in adhesion, internalization, and toxicity associated with ClpC allowed us to assess intracellular persistence without complicating circumstances. To do this, intracellular bacterial cell densities were determined at 24 h, 96 h, and 168 h post-infection of HaCaT and EA.hy926 cells (Fig. 3). Within 24 h of internalization, HaCaT cells killed approximately 75% of the intracellular bacteria, irrespective of the presence of ClpC (Figs 3a, b). Similar results were obtained with EA.hy926 cells (Figs 3c, d). Interestingly, after 96 h and 168 h of cultivation, significantly greater numbers of viable bacteria were present in both HaCaT (5.2-fold and 20.3-fold, respectively) and EA.hy926 cells (3.2-fold and 24.9-fold, respectively) infected with the *clpC* mutant strain PBM001 relative to those infected with the wild-type strain. Complementation of *clpC* restored persistence to near wild-type levels (Fig. 3).

**Figure 4:**
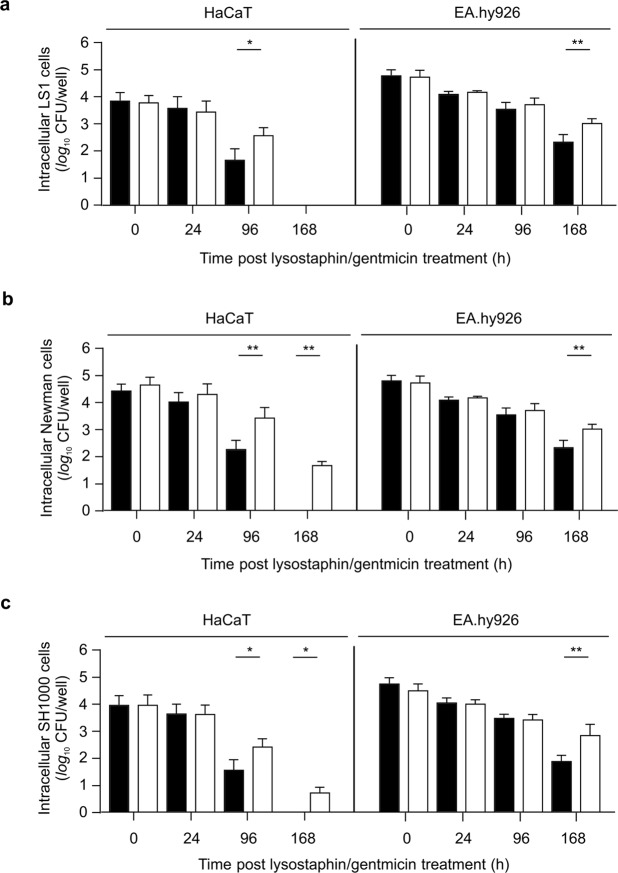
Impact of the strain background on the ClpC effect on intracellular survival of *S. aureus* in non-professional phagocytes. HaCaT and EA.hy926 cells were infected with the *S. aureus* strains LS1 (**a**), Newman (**b**), SH1000 (**c**) (WT; black bars), and the isogenic *clpC* mutants (*clpC*; white bars) at a MoI of 100, respectively, and co-cultured for 90 min. Extracellular and adherent bacteria were subsequently removed by washing and lysostaphin/gentamicin treatment, and the infected cell lines cultured for up to 168 h in cell culture medium supplemented with gentamicin. At the time points indicated, cells were removed, lysed by sonication, and surviving bacteria in lysates determined by plate counting. Data are presented as mean + SD (*n* = 6 biological replicates). *P < 0.05; **P < 0.01 (Mann-Whitney *U* test between WT and isogenic *clpC* mutant at a given time point). TpL/G, time post lysostaphin/gentamicin treatment.

The ability of *S. aureus* to persist in non-professional phagocytes can be affected by the bacterial genetic background^4^. To exclude that the intracellular survival phenotype attributed to ClpC is unique to strain DSM20231, the *clpC* mutation was transduced into *S. aureus* strains LS1, Newman, and SH1000, and persistence was assessed. Irrespective of the genetic background, inactivation of *clpC* did not alter internalization of *S. aureus* (Fig. 4) or survival during the first 24 h post-infection. At 96 h post-infection all *clpC* mutants maintained significantly greater bacterial numbers than did the isogenic wild-type strains in HaCaT, while significantly increased cell numbers were observed in EA.hy926 cells after 168 h post-infection. Taken together, these data demonstrate that *clpC* inactivation enhances persistence in keratinocytes and endothelial cells and is unrelated to the strain background. These observations are consistent with ClpC activity being greatest late in the growth cycle of *S. aureus*^9^.

### SCV formation by intracellularly persisting *S. aureus* is independent of ClpC

Earlier work demonstrated that intracellular passage of *S. aureus* in eukaryotic cells enhances SCV formation^6,18^. To assess whether ClpC enhances persistence of *S. aureus* via augmentation of SCV formation, we determined the ratios of SCVs formed in EA.hy926 cells after 168 h post-infection (Fig. S2). Consistent with other *S. aureus* genetic lineages^6,18^, strain DSM20231 produced SCVs upon intracellular passage (3.3 ± 1.5% of colonies with a colony area ≤10 than the average colony area of normal growing colonies). The *clpC* mutant PBM001 produced a slightly higher ratio of SCVs upon intracellular passage (4.5 ± 1.4%); however, this difference was not statistically significant (*P* = 0.242, Mann-Whitney *U* test). These findings suggest that the increased intracellular survival capacity of PBM001 in non-professional phagocytic cells is not due to an increased ability to form SCVs.

### ClpC enhanced intracellular survival of *S. aureus* requires MazEF

**Figure 5:**
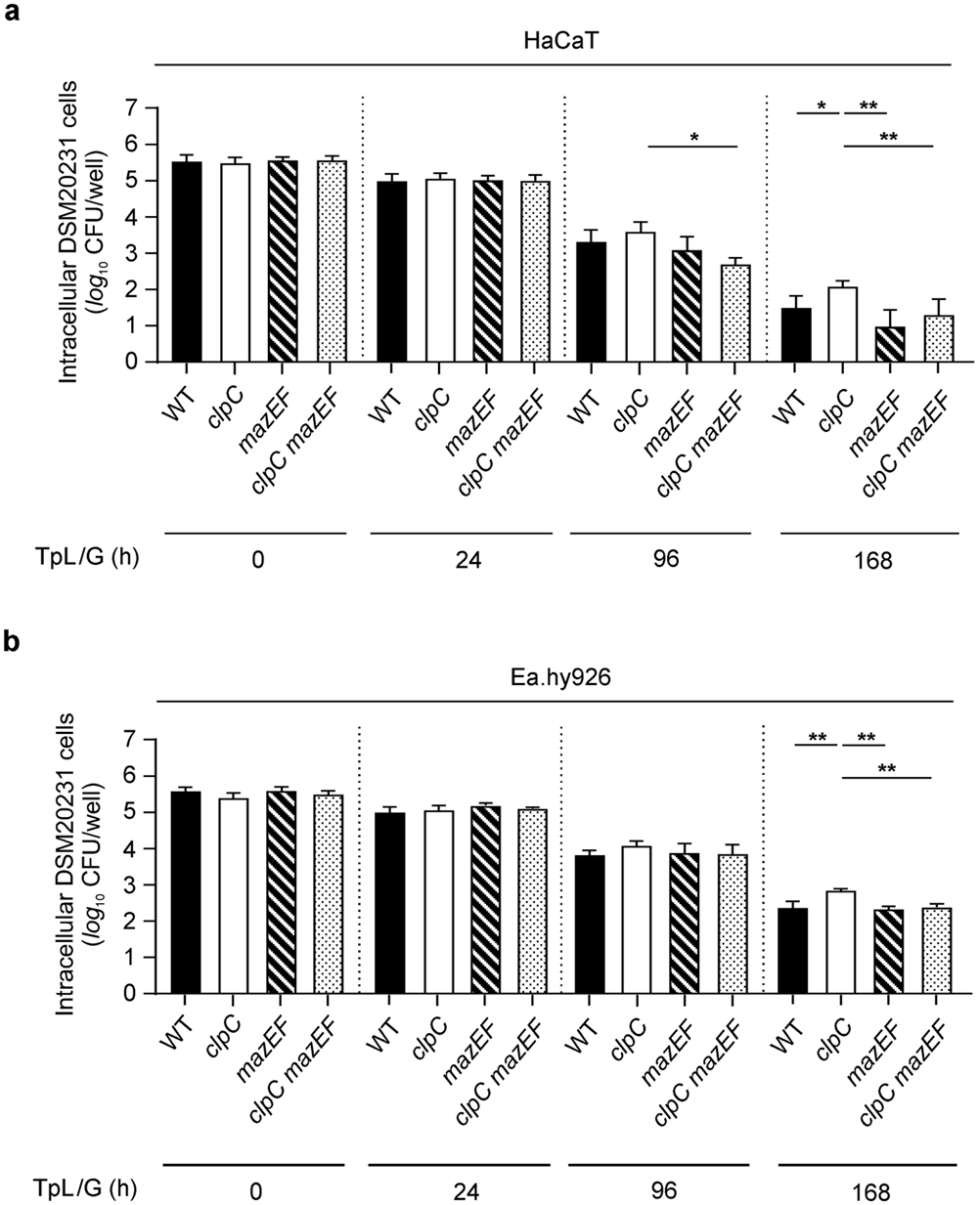
Impact of MazEF on the ClpC effect on intracellular survival of *S. aureus* DSM20231 in keratinocytes and endothelial cells. HaCaT (**a**) and EA.hy926 (**b**) cells were infected with the *S. aureus* strains DSM20231 (WT; black bars), PBM001 (*clpC*; white bars), DSM20231 *mazEF* (*mazEF*; transversal dashed bars), and DSM20231 *clpC mazEF* (*clpC mazEF*; dotted bars) at a MoI of 100, respectively, and co-cultured for 90 min. Extracellular and adherent bacteria were subsequently removed by washing and lysostaphin/gentamicin treatment, and the infected cell lines cultured for up to 168 h in cell culture medium supplemented with gentamicin. At the time points indicated, cells were removed, lysed by sonication, and surviving bacteria in lysates determined by plate counting. Data are presented as mean + SD (*n* = 6–8 biological replicates). *P < 0.05; **P < 0.01 (Kruskal Wallis test followed by Dunn’s post-hoc test between WT and mutants at a given time point). TpL/G, time post lysostaphin/gentamicin treatment.

ClpCP modulates the activity of the MazEF toxin/antitoxin system by degrading the antitoxin, MazE, in a growth phase-dependent manner^12,13^. Additionally, MazEF alters the intracellular survival of *S. aureus* in osteoblasts^15^, suggesting that ClpC might affect the intracellular survival of *S. aureus* through MazEF. To test this hypothesis, we created *mazEF* mutants in strains DSM20231 and PBM001 and assessed their ability to survive intracellularly (Fig. 5). Similar to strain DSM20231, all three derivatives had comparable numbers of internalized bacteria after 90 min of incubation with HaCaT and Ea.hy926 cells. As expected, we observed that strain PBM001 had significantly increased numbers of viable bacteria in HaCaT (Fig. 5a) or Ea.hy926 (Fig. 5b) cells after 168 h of cultivation relative to the wild-type strain. Deletion of *mazEF* in DSM20231 did not markedly alter the invasion and intracellular survival of the mutant in keratinocytes and endothelial cells. At 168 h post lysostaphin/gentamicin treatment, the *mazEF* deletion strain had reduced numbers of intracellularly surviving bacteria in HaCaT cells, however this was not statistical significant (*P* = 0.124, Mann-Whitney *U* test between WT and *mazEF* mutant). These findings are in contrast to those made by Kolenda and colleagues^15^, where deletion of *mazEF* in strain HG003 enhanced intracellular survival in osteoblasts at 24 and 48 h post infection. Interestingly, in the *clpC mazEF* double mutant, the numbers of surviving bacteria were equivalent to those of strain DSM20231 at all time points, indicating that PBM001 required an intact *mazEF* locus to augment its intracellular persistence in HaCaT and Ea.hy926 cells. Taken together, these data suggest that ClpC influences *S. aureus* intracellular survival in keratinocytes and endothelial cells, in part, via the modulation of MazEF activity.

### Inactivation of *clpC* alters the transcription of *hla*, *RNAIII*, and *spa in vitro* and in internalized bacteria

**Figure 6:**
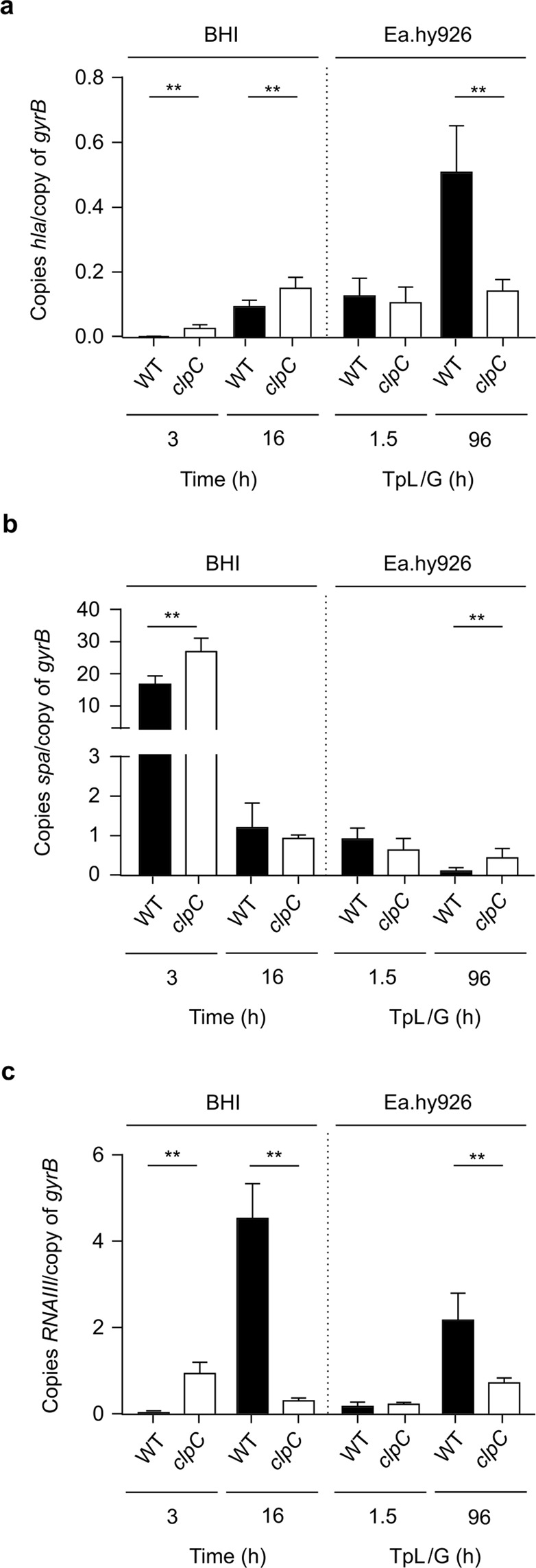
Impact of ClpC on the transcription of *hla*, *spa*, and *RNAIII*. Quantitative transcript analyses of *hla* (**a**), *spa* (**b**), and *RNAIII* (**c**) by qRT-PCR in DSM20231 (WT, black bars) and PBM001 (*clpC*, white bars) cells grown in BHI to the time points indicated or persisting intracellularly in Ea.hy926 cells for 90 min and 96 h, respectively. Transcripts were quantified in reference to the transcription of gyrase B (in copies per copy of *gyrB*). Data are presented as mean + SD (*n* = 5–6 biological replicates). **P < 0.01 (Mann-Whitney *U* test between WT and mutant at a given time point). TpL/G, time post lysostaphin/gentamicin treatment.

The pore-forming cytolysin α-toxin (encoded by *hla*) is an important virulence factor of *S. aureus* that is under multiple levels of control^19^. Deletion of *hla* in *S. aureus* enhances intracellular persistence in HaCaT cells^20^, and it inhibits apoptosis^21^. Interestingly, *hla* mRNA stability is decreased by MazF activity in strain Newman^22^, which is in part controlled by the ClpCP proteolytic complex^13^. Similarly, the stability of *spa* mRNA (protein A) is also affected by MazF activity in different *S. aureus* strains (Newman and HG003)^22,23^. These observations are important because transcription of both *hla* and *spa* are altered in SCVs and persister cells^2^. To assess if ClpC alters transcription of *hla* and *spa*, we used qRT-PCR to determine the mRNA levels of *hla* and *spa* in strains DSM20231 and PBM001 both *in vitro* and after host cell invasion (Fig. 6). Consistent with previous observations^24^, during batch cultivation transcription of *hla* and *spa* was growth phase-dependent in strain DSM20231. Specifically, *hla* mRNA levels were most abundant late in the growth cycle (*i.e*., after 16 h of cultivation), while *spa* mRNA levels were elevated early in the growth cycle (*i.e*., after 3 h of cultivation). Similarly, transcription of *hla* and *spa* were growth phase dependent in the *clpC* mutant, although, *hla* and *spa* mRNA levels were greater in the *clpC* mutant relative to the wild-type strain. This latter observation is consistent with ClpCP affecting the stability of *hla* and *spa* mRNAs via degradation of the antitoxin MazE. To assess the *hla* and *spa* mRNA levels in internalized bacteria, EA.hy926 cells were infected and total RNA was isolated and used for qRT-PCR. At 90 minutes post-infection, *hla* and *spa* mRNA levels from internalized DSM20231 and PBM001 cells were equivalent between the wild-type and the *clpC* mutant. In contrast, after 96 h in EA.hy926 cells, *clpC* inactivated bacteria had significantly higher levels of *spa* mRNA, consistent with the hypothesis that ClpC affects transcription of *spa* via the control of MazEF activity. However, for *hla* mRNA levels, significantly more *hla* mRNA was found in DSM20231 cells that persisted for 96 h in EA.hy926 cells relative to that found in the *clpC* mutant (Fig. 6a). Although this observation is consistent with the reduced survival of intracellular DSM20231 cells (Fig. 3), it is inconsistent with the indication that ClpC controls *hla* transcription primarily via the modulation of MazEF activity. That being said, since transcription of *hla* is under multiple levels of control^19^, it cannot be excluded that ClpC might promote *hla* transcription under these conditions via cooperation with other regulatory elements, such as CcpA, CodY, Rex, and AgrA^25–28^, all of which are substrates for ClpC in *S. aureus*^29^. One of the regulatory factors promoting *hla* transcription and being affected by ClpC is the response regulator AgrA of the *agr* locus^27,29^. To determine whether ClpC alters transcription of the *agr* locus, we assessed the transcript levels of *RNAIII*, the main effector molecule of the *agr* locus^30^, in strains DSM20231 and PBM001 in *vitro* and after host cell invasion (Fig. 6c). Consistent with previous observations, transcription of *RNAIII* was growth phase-dependent^31^. In batch culture grown DSM20231 cells, *RNAIII* transcript levels were low during exponential growth (3 h), but increased in the later growth phase (16 h). In PBM001 cells, the *clpC* mutant produced a significantly greater amount of *RNAIII* in the exponential growth phase, relative to that of wild-type bacteria. In contrast to the exponential growth phase level of *RNAIII*, after 16 h of cultivation, PBM001 cells had very little *RNAIII*. In short-time internalized bacteria (i.e. after 90 minutes post-infection), *RNAIII* levels in DSM20231 and PBM001 cells were equivalent. In contrast, significantly more *RNAIII* was found in DSM20231 cells that persisted for 96 h in EA.hy926 cells relative to that found in the *clpC* mutant strain (Fig. 6c), suggesting that ClpC enhances transcription of *hla* in intracellularly persisting DSM20231 cells. The simplest explanation for these data is that ClpC enhances transcription of the *agr* locus, causing an increase in *RNAIII* transcription, which in turn increases *hla* transcription. However, alternative regulatory circuits cannot be excluded.

### Concluding remarks

The ability of *S. aureus* to invade host cells and to persist intracellularly is considered a major contributor to its ability to cause chronic diseases. We show here that inactivation of *clpC* in *S. aureus* augments the long-term intracellular survival in non-professional phagocytes, suggesting that ClpC modulates intracellular persistence. This is likely mediated, at least in part, due to an interaction of ClpC with AgrA, and to the ClpCP mediated degradation of MazE. Degradation of MazE activates the site-specific endoribonuclease MazF^13^, thereby inducing bacterial cell stasis^22^.

## Materials and Methods

### Bacterial strains, plasmids and culture conditions

Eukaryotic cell lines, bacterial strains, and plasmids used in this study are listed in Table 1. Bacteria were routinely grown at 37 °C with 230 rpm of aeration in Brain Heart Infusion (BHI) broth or Luria Bertani (LB) broth purchased from Becton Dickinson (Heidelberg, Germany), using a culture medium-to-flask volume of 1:10. Where required, media were supplemented with (per milliliter): 10 µg erythromycin, or 10 µg tetracycline. HaCaT cells were cultured in MCDB153 Basal Medium (Biochrom AG, Berlin, Germany), 10% fetal bovine serum (FBS; PAA, Cölbel, Germany), and 1% non-essential amino acids (NEAA; Gibco, Darmstadt, Germany). EA.hy926 cells were cultivated in Dulbecco´s Modified Eagle Medium (Fisher Scientific, Schwerte, Germany) supplemented with 10% FBS. Both cell lines were grown in a humidified atmosphere at 37 °C and 5% CO_2_.

**Table 1:** Strains and plasmids used in this study.

Strain/cell line	Relevant genotype and phenotype^a^	Reference or source
**Cell lines**		
EA.hy926	Hybrid cell line derived by fusing human umbilical vein endothelial cells with the permanent human cell line A549	^35^
HaCaT	‘Human adult low calcium high temperature keratinocytes’, spontaneously immortalized human keratinocyte cell line	^36^
***S. aureus***		
DSM20231	Serovar 3 type strain, ATCC12600	^37^
DSM20231 *mazEF*	DSM20231 *mazEF::lox72*	This study
DSM20231 *mazEF clpC*	DSM20231 *mazEF::lox72 clpC::erm*(B); Em^R^	This study
LS1	Murine arthritis isolate	^38^
LS1 *clpC*	LS *clpC::erm*(B); Em^R^	This study
Newman	Clinical isolate (ATCC 25904)	^39^
Newman *clpC*	Newman *clpC::erm*(B); Em^R^	This study
PBM001	DSM20231 *clpC::erm*(B); Em^R^	^32^
PBM001 *clpC*^+^	PBM001 *clpC::*pBT_*SACOL0570*’, *clpC*^+^; Tc^R^	This study
RN4220	Restriction negative, modification positive NCTC 8325-4 derivative	^40^
SH1000	NCTC 8325-4 *rsbU*^+^ derivative	^41^
SH1000 *clpC*	SH1000 *clpC::erm*(B); Em^R^	This study
**Plasmids**		
pBT	pBC-SK derivative harboring an 1.6-kb PCR fragment of *tet*(L) from pHY300PLK cloned into the *Alw*26I restriction site; Tc^R^	^33^
pBT_*clpC’*	pBT derivative harboring an 1-kb DNA fragment of the C-terminus of *clpC*; Tc^R^	This study
pBT2-*mazEF*-ko-*ermB*	pBT2 derivative harboring the P_mazEF_ promoter and the transcriptional terminator downstream of *mazEF* and a lox flanked erythromycin resistance cassette replacing the *mazE* and *mazF* open reading frames.	^23^

^a^Abbreviations are as follows: Ap^r^, ampicillin resistant; Em^r^, erythromycin resistant; Km^r^, kanamycin resistant; Tc^r^, tetracyclin resistant.

### Construction of *clpC* and *mazEF* mutants

*S. aureus clpC* mutants were obtained by transducing the resistance cassette tagged *clpC* mutation of PBM001^32^ into strains LS1, Newman, SH1000, and DSM20231 *mazEF*, using phage 85. The DSM20231 *mazEF* mutant was created using plasmid pBT2-*mazEF-ko-ermB* as described^23^. For *cis*-complementation of PBM001 with a functional *clpC*, a 1-kb DNA fragment containing the C-terminus of *clpC*, including the terminator sequence, was amplified by PCR from the chromosomal DNA of *S. aureus* strain N315 using the primer pair MBH325/MBH326 (Table 2). The resulting PCR product was digested with *Kpn*I/*Xho*I, and subsequently cloned into the *Kpn*I/*Xho*I-digested vector pBT^33^ to generate the suicide plasmid pBT_*clpC’*. The plasmid was transformed into *S. aureus* strain RN4220 and selected for tetracycline-resistance. A tetracycline-resistant RN4220 derivative that integrated pBT_*clpC’* in its chromosome at the *clpC* locus was used as donor to transduce the *tet*(L)-tagged *clpC* allele into PBM001, thereby replacing the *erm*(B)-tagged *clpC* mutation with the *tet*(L)-tagged *clpC* allele. Restoration of the *clpC* wild-type gene in one of the transductants, PBM001 *clpC*^+^, was verified by DNA sequencing.

**Table 2:** Primers used in this study.

Primer	Sequence (5′-3′)^a^
**Cloning primer**	
MBH325	gtcggtacCGGATTACTTATTTCTGTTATGG (*Kpn*I)
MBH326	ggtctcgaGATTCTAATTTTGTATGTCTTGATTG (*Xho*I)
**qRT-PCR primer**	
*gyrB* for	GACTGATGCCGATGTGGA
*gyrB* rev	AACGGTGGCTGTGCAATA
*hla* for	AACCCGGTATATGGCAATCAACT
*hla* rev	CTGCTGCTTTCATAGAGCCATTT
*RNAIII* for	AGGAGTGATTTCAATGGCACAAG
*RNAIII* rev	TGTGTCGATAATCCATTTTACTAAGTC
*spa* for	TGCTGACAAAATTGCTGCAGATA
*spa* rev	GCATGGTTTGCTGGTTGCTT

^a^Restriction sites are underlined and specified in parentheses. Linker nucleotides are given as small letters.

### Single cell force spectroscopy

Adhesion of DSM20231 and its isogenic *clpC* mutant (PBM001) to endothelial cells was determined as follows: EA.hy926 cells (7.5 × 10^4^ cells/petri dish) were seeded in glass-bottomed petri dishes (World Precision Instruments, Berlin, Germany) and allowed to adhere for 24 h in supplemented DMEM. Single, stationary phase cells (16 h of growth in LB), of strains DSM20231 and PBM001 were attached to tipless cantilevers (MLCT-0; Bruker Nano, Santa Barbara, CA) and tested for viability as described^17^. Cantilevers were calibrated before each set of experiments, and bacterial probes were used to measure the interaction forces between the bacterial cell and Ea.hy926 cells at room temperature by recording 8 × 8 force-distance curves on 1 × 1 µm lamellipodial areas of the cells. Force-distance curves were obtained in DMEM at room temperature with a Bioscope Catalyst atomic force microscope (Bruker) with a ramp size of 7 µm, a ramp velocity of 1 µm/s, a force trigger of 1 nN, and a surface delay time of 250 ms. Data were analyzed using the Nanoscope Analysis software (Bruker).

### Adhesion, invasion and persistence assay

The adhesion, internalization and persistence assays were carried out as described^34^, with minor modifications. Briefly, EA.hy926 and HaCaT cells were grown for 24 hours in 24-well plates until confluent (~4.5 × 10^5^ cells per well). Bacteria from overnight cultures were harvested by centrifugation, washed, and suspended in phosphate-buffered saline (PBS; Gibco). Eukaryotic cells were inoculated with bacteria using a multiplicity of infection (MoI) of either 1, 10, or 100, centrifuged for 1 min at 1000 × g to promote attachment of bacteria to the cells, and subsequently co-cultivated for 90 min at 37 °C. Thereafter, wells were washed three times with PBS to remove unbound bacteria. Eukaryotic cells were then detached by adding 0.5% Trypsin (Biochrom AG), and lysed by sonication at 50 W for 15 s in H_2_O, and colony-forming units (CFUs) were determined by plating dilutions of the lysates on sheep blood agar (BD). For the lysostaphin/gentamicin protection assays (internalization), adherent but not internalized bacterial cells were subsequently removed by incubating the bacteria-challenged cells for 1 h at 37 °C in appropriate culture media supplemented with 20 µg/ml lysostaphin (Genmedics, Reutlingen, Germany) and 100 µg/ml gentamicin (Refobacin, Merck, Darmstadt, Germany). The eukaryotic cells were washed in PBS to remove the antimicrobials. Wells without eukaryotic cells were routinely run in parallel to control the efficacy of the lysostaphin/gentamicin treatment. Eukaryotic cells were subsequently lysed and CFU rates in lysates determined as described. For long-term survival experiments, infected eukaryotic cells were cultivated in the presence of gentamicin (100 µg/ml), lysostaphin (20 µg/ml), and 2% FCS for up to 168 h. After 96 h, cell cultures were supplied with fresh media, 10% FCS, and antibiotics. Numbers of intracellular surviving bacteria were determined after 24, 96, and 168 h post lysostaphin/gentamicin treatment as described.

### Cytotoxicity assay

EA.hy926 and HaCaT cells were seeded and infected with bacteria as described. Following the lysostaphin/gentamicin treatment, cells were washed once with the appropriate culture medium, then cultured for 24 h in fresh cell culture medium without antibiotics. Cells were detached by trypsinization and stained with 1 mg/mL propidium iodide (PI; Fluka, Buchs, Switzerland) for 10 min. Stained cells were washed with PBS, and the PI-staining of cells analyzed by fluorescence-activated cell sorting (FACS, CellQuestPro, Becton Dickinson, Franklin Lakes, NJ, USA).

### Cell viability assay

EA.hy926 and HaCaT cells were grown for 24 hours in 96-well plates until confluent (~8 × 10^4^ cells per well). Cell cultures were infected with bacteria, non-internalized bacteria subsequently removed, and infected eukaryotic cells cultivated as described in the persistence assay. Bacteria-free eukaryotic cell cultures served as controls. After 96 h and 168 h, respectively, 100 µl aliquots of a freshly prepared 3-(4.5-dimethylthiazol-2-yl)-2.5-diphenyltetrazolium bromide (MTT; Merck) solution (2.5 mg/ml MTT dissolved in PBS, pH 7.2) were added to the wells, and cell cultures were incubated for additional 2 h at 37 °C and 5% CO_2_. Medium and MTT solution were subsequently removed and cells solubilized in 100 µl of extraction buffer (20% sodium dodecyl sulfate, 30% dimethylformamide, 2% acetic acid, pH 4.7) for 3 h in the dark at room temperature and 50 rpm. Formazan formation was quantified with an EnSight multimode plate reader and the Kaleido data acquisition and analysis software (PerkinElmer, Rodgau, Germany) by measuring the light absorbance at 570 nm and a reference wavelength of 630 nm.

### qRT-PCR analyses

Quantification of mRNA for *in vitro* cultivated bacteria by real-time reverse transcription PCR (qRT-PCR) was carried out as described^8^. For the quantification of mRNA in intracellularly persisting bacteria by qRT-PCR, the following protocol was used: EA.hy926 cells were infected with bacteria as described. After 90 min and 96 h post lysostaphin/gentamicin treatment, respectively, Ea.hy926 cells were washed once with PBS and subsequently lysed with ice-cold H_2_O supplemented with RNA protect (Qiagen, Hilden, Germany) by pipetting. Lysates from 5 wells were pooled and centrifuged for 15 min at 5600 × g and 4 °C. Cell pellets were suspended in ice-cold TE (10 mM Tris/HCl, 1 mM EDTA pH 8.0) and bacteria were disrupted, total RNA isolated, and qRT-PCRs carried out as described^8^ using the primers listed in Table 2. The level of mRNA was normalized against the internal control *gyrB*. Transcript amounts were expressed as the fold difference relative to the control (2^ΔΔCT^ where ΔCT represents the difference in threshold cycle between the target and control genes).

### Statistical analyses

The statistical significance of changes between groups was assessed with Kruskal Wallis tests followed by Dunn’s post-hoc tests (3 and more groups) or the Mann-Whitney *U* test (two groups) using the GraphPad software package Prism 6.01. *P* values < 0.05 were considered significant.

## Supplementary information

Supplementary information

## Data Availability

The datasets generated during and/or analyzed during the current study are available from the corresponding author on reasonable request.

## References

[1] Lowy FD (1998). *Staphylococcus aureus* infections. The New England Journal of Medicine.

[2] Proctor RA (2014). *Staphylococcus aureus* Small Colony Variants (SCVs): a road map for the metabolic pathways involved in persistent infections. Frontiers in Cellular and Infection Microbiology.

[3] Sinha B, Fraunholz M (2010). *Staphylococcus aureus* host cell invasion and post-invasion events. International Journal of Medical Microbiology.

[4] Strobel M (2016). Post-invasion events after infection with *Staphylococcus aureus* are strongly dependent on both the host cell type and the infecting *S. aureus* strain. Clinical Microbiology and Infection.

[5] von Eiff C, Peters G, Becker K (2006). The small colony variant (SCV) concept–the role of staphylococcal SCVs in persistent infections. Injury.

[6] Tuchscherr L (2015). Sigma factor SigB is crucial to mediate *Staphylococcus aureus* adaptation during chronic infections. PLoS Pathogens.

[7] Chatterjee I, Herrmann M, Proctor RA, Peters G, Kahl BC (2007). Enhanced post-stationary-phase survival of a clinical thymidine-dependent small-colony variant of *Staphylococcus aureus* results from lack of a functional tricarboxylic acid cycle. Journal of Bacteriology.

[8] Chatterjee I (2005). *Staphylococcus aureus* ClpC is required for stress resistance, aconitase activity, growth recovery, and death. Journal of Bacteriology.

[9] Chatterjee I (2009). *Staphylococcus aureus* ClpC ATPase is a late growth phase effector of metabolism and persistence. Proteomics.

[10] Mashruwala AA (2019). The ClpCP complex modulates respiratory metabolism in *Staphylococcus aureus* and is regulated in a SrrAB-dependent manner. Journal of Bacteriology.

[11] Luong TT (2011). *Staphylococcus aureus* ClpC divergently regulates capsule via *sae* and *codY* in strain Newman but activates capsule via *codY* in strain UAMS-1 and in strain Newman with repaired *saeS*. Journal of Bacteriology.

[12] Donegan NP, Marvin JS, Cheung AL (2014). Role of adaptor TrfA and ClpPC in controlling levels of SsrA-tagged proteins and antitoxins in *Staphylococcus aureus*. Journal of Bacteriology.

[13] Donegan NP, Thompson ET, Fu Z, Cheung AL (2010). Proteolytic regulation of toxin-antitoxin systems by ClpPC in *Staphylococcus aureus*. Journal of Bacteriology.

[14] Aizenman E, Engelberg-Kulka H, Glaser G (1996). An *Escherichia coli* chromosomal “addiction module” regulated by guanosine [corrected] 3’,5’- bispyrophosphate: a model for programmed bacterial cell death. Proceedings of the National Academy of Sciences of the United States of America.

[15] Kolenda C, Josse J, Sierra R, Renzoni A, Laurent F (2017). Importance of MazEF toxin-antitoxin system for intracellular development of *Staphylococcus aureus* in osteoblasts. Orthopaedic Proceedings.

[16] Frees D (2004). Clp ATPases are required for stress tolerance, intracellular replication and biofilm formation in *Staphylococcus aureus*. Molecular Microbiology.

[17] Thewes N (2015). A detailed guideline for the fabrication of single bacterial probes used for atomic force spectroscopy. The European Physical Journal. E, Soft matter.

[18] Vesga O (1996). *Staphylococcus aureus* small colony variants are induced by the endothelial cell intracellular milieu. The Journal of Infectious Diseases.

[19] Bischoff Markus, Romby Pascale (2016). Genetic Regulation. Staphylococcus: Genetics and Physiology.

[20] Soong G (2015). Methicillin-resistant *Staphylococcus aureus* adaptation to human keratinocytes. mBio.

[21] Menzies BE, Kourteva I (2000). *Staphylococcus aureus* alpha-toxin induces apoptosis in endothelial cells. FEMS Immunology and Medical Microbiology.

[22] Fu Z, Tamber S, Memmi G, Donegan NP, Cheung AL (2009). Overexpression of MazFsa in *Staphylococcus aureus* induces bacteriostasis by selectively targeting mRNAs for cleavage. Journal of Bacteriology.

[23] Schuster CF (2015). The MazEF toxin-antitoxin system alters the beta-lactam susceptibility of *Staphylococcus aureus*. PloS one.

[24] Cheung AL, Bayer AS, Zhang G, Gresham H, Xiong YQ (2004). Regulation of virulence determinants in vitro and *in vivo* in *Staphylococcus aureus*. FEMS Immunology and Medical Microbiology.

[25] Majerczyk CD (2008). *Staphylococcus aureus* CodY negatively regulates virulence gene expression. Journal of Bacteriology.

[26] Seidl K (2006). *Staphylococcus aureus* CcpA affects virulence determinant production and antibiotic resistance. Antimicrobial Agents and Chemotherapy.

[27] Liang X (2011). Identification of single nucleotide polymorphisms associated with hyperproduction of alpha-toxin in *Staphylococcus aureus*. PloS one.

[28] Somerville GA, Proctor RA (2009). At the crossroads of bacterial metabolism and virulence factor synthesis in Staphylococci. Microbiology and Molecular Biology Reviews.

[29] Graham JW, Lei MG, Lee CY (2013). Trapping and identification of cellular substrates of the *Staphylococcus aureus* ClpC chaperone. Journal of Bacteriology.

[30] Janzon L, Arvidson S (1990). The role of the delta-lysin gene (*hld*) in the regulation of virulence genes by the accessory gene regulator (*agr*) in *Staphylococcus aureus*. The EMBO Journal.

[31] Janzon L, Lofdahl S, Arvidson S (1989). Identification and nucleotide sequence of the delta-lysin gene, *hld*, adjacent to the accessory gene regulator (*agr*) of *Staphylococcus aureus*. Molecular & General Genetics.

[32] Becker P, Hufnagle W, Peters G, Herrmann M (2001). Detection of differential gene expression in biofilm-forming versus planktonic populations of *Staphylococcus aureus* using micro-representational-difference analysis. Applied and Environmental Microbiology.

[33] Giachino P, Engelmann S, Bischoff M (2001). Sigma(B) activity depends on RsbU in *Staphylococcus aureus*. Journal of Bacteriology.

[34] Bur S, Preissner KT, Herrmann M, Bischoff M (2013). The *Staphylococcus aureus* extracellular adherence protein promotes bacterial internalization by keratinocytes independent of fibronectin-binding proteins. The Journal of Investigative Dermatology.

[35] Edgell CJ, McDonald CC, Graham JB (1983). Permanent cell line expressing human factor VIII-related antigen established by hybridization. Proceedings of the National Academy of Sciences of the United States of America.

[36] Boukamp P (1988). Normal keratinization in a spontaneously immortalized aneuploid human keratinocyte cell line. The Journal of Cell Biology.

[37] Silvestri LG, Hill LR (1965). Agreement between deoxyribonucleic acid base composition and taxometric classification of Gram-positive cocci. Journal of Bacteriology.

[38] Bremell T, Abdelnour A, Tarkowski A (1992). Histopathological and serological progression of experimental *Staphylococcus aureus* arthritis. Infection and Immunity.

[39] Duthie ES (1952). Variation in the antigenic composition of staphylococcal coagulase. Journal of General Microbiology.

[40] Kreiswirth BN (1983). The toxic shock syndrome exotoxin structural gene is not detectably transmitted by a prophage. Nature.

[41] Horsburgh MJ (2002). SigmaB modulates virulence determinant expression and stress resistance: characterization of a functional *rsbU* strain derived from *Staphylococcus aureus* 8325-4. Journal of Bacteriology.

