# Supplementary material for: ClpC affects the intracellular survival capacity of Staphylococcus aureus in non-professional phagocytic cells
Source: Sci Rep. 2019 Nov 7;9:16267. doi: 10.1038/s41598-019-52731-3 (PMC6838064; doi:10.1038/s41598-019-52731-3)
Supplement: Supplementary file 1 — Supplementary information [file 41598_2019_52731_MOESM1_ESM.pdf]

Supplementary Information

ClpC affects the intracellular survival capacity of *Staphylococcus aureus* in non-professional phagocytic cells

Gubesh Gunaratnam[1], Lorena Tuchscherr[2], Mohamed I. Elhawy[1], Ralph Bertram[3], Janina Eisenbeis[1], Christian Spengler[4], Thomas Tschernig[5], Bettina Löffler[2], Greg A. Somerville[6], Karin Jacobs[4], Mathias Herrmann[1,7], and Markus Bischoff[1*]

[1]Institute of Medical Microbiology and Hygiene, Saarland University, Homburg/Saar, Germany

[2]Institute of Medical Microbiology, Jena University Hospital, Jena, Germany

[3]Institute of Clinical Hygiene, Medical Microbiology and Infectiology, Paracelsus Medical University, Nuremberg, Germany

[4]Experimental Physics, Saarland University, Saarbrucken, Germany

[5]Institute of Anatomy and Cell Biology, Saarland University, Homburg/Saar, Germany

[6]School of Veterinary Medicine and Biomedical Sciences, University of Nebraska-Lincoln, Lincoln, Nebraska, USA

[7]Institute of Medical Microbiology, University Hospital of Münster, Münster, Germany

Correspondence:

Dr. Markus Bischoff

markus.bischoff@uks.eu

1. Adhesion forces between S. *aureus* and the extracellular matrix of endothelial cells

	WT			Δ*clpC*		
	**P_{adh} (%)**	**F_{adh} (pN)**	**L_{rupt} (nm)**	**P_{adh} (%)**	**F_{adh} (pN)**	**L_{rupt} (nm)**
cell 1	98	204 ± 93	1898 ± 1759	87	142 ± 87	1124 ± 1132
cell 2	91	193 ± 128	1579 ± 2060	98	296 ± 118	2399 ± 1360
cell 3	84	149 ± 82	1420 ± 1166	84	87 ± 65	1317 ± 1444
cell 4	96	185 ± 83	1920 ± 1281	95	91 ± 64	1235 ± 1252
cell 5	92	144 ± 120	1679 ± 1706	100	184 ± 98	1414 ± 1041
**mean**	**92 ± 6**	**172 ± 104**	**1688 ± 1594**	**93 ± 7**	**163 ± 117**	**1507 ± 1327**

Table S1: Probability of adhesion (P_{adh} = % of curves with measurable adhesion forces), maximum adhesion force (F_{adh}), and rupture length (L_{rupt}) measured between single DSM20231 (WT) or PBM001 (Δ*clpC*) bacteria and extracellular matrix of sessile Ea.hy926 cells (64 measurements per cell pair recorded on a 1 x 1 µm areal on the lamellipodial region of the endothelial cell).

2. Impact of ClpC on the cell viability of intracellularly *S. aureus* infected non-professional phagocytic cells

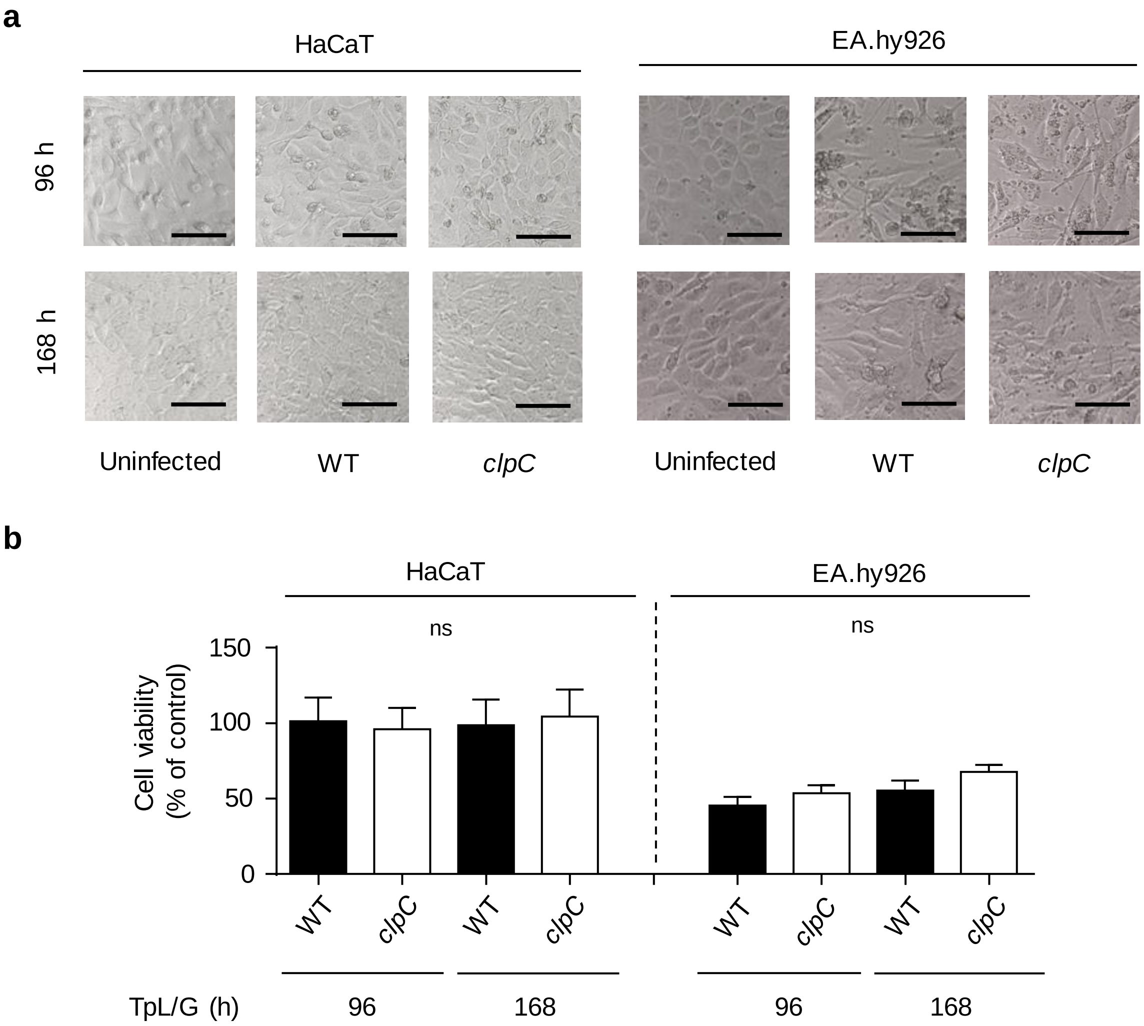

Figure S1: HaCaT and EA.hy926 cells were seeded on 96 well plates, infected with the *S. aureus* strains DSM20231 (WT; black bars), and the isogenic *clpC* mutant PBM001 (*clpC*; white bars) at a MoI of 100, respectively, and co-cultured for 90 min. Extracellular and adherent bacteria were subsequently removed by washing and lysostaphin/gentamicin treatment. Infected eukaryotic cells were cultured for up to 168 h as described in Methods. Bacteria-free cell cultures served as control **a:** Representative light microscopy images of uninfected and infected cell cultures at 96 h and 168 h post lysostaphin/gentamicin treatment. Scale bar = 50 µm. **b:** 96 h and 168 h old bacteria-free and intracellularly *S. aureus* infected eukaryotic cell cultures were subjected to MTT-based cell viability assays as described in Methods. Values were normalized to the signals seen with the bacteria-free controls at a given time, which were set to 100%. Data are presented as mean + SD (*n*=4 biological replicates). ns, not significant (Mann-Whitney *U* test between WT and mutant at a given time point). TpL/G, time post lysostaphin/gentamicin treatment.

3. Impact of ClpC on SCV formation of intracellularly persisting *S. aureus* cells

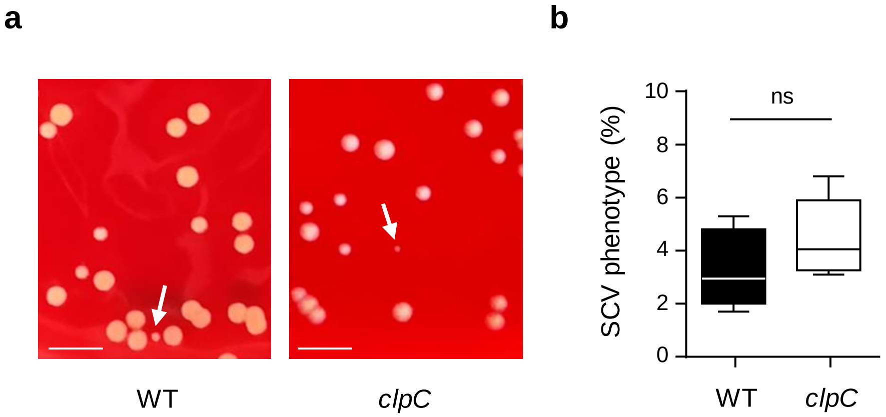

Figure S2: EA.hy926 cells were infected with the *S. aureus* strains DSM20231 (WT; black bars), and the isogenic *clpC* mutant PBM001 (*clpC*; white bars) at a MoI of 100, respectively, and co-cultured for 90 min. Extracellular and adherent bacteria were subsequently removed by washing and lysostaphin/gentamicin treatment, and the infected endothelial cells cultured for 168 h in cell culture medium supplemented with gentamicin. At the end of the incubation time, Ea.hy926 cells were detached by trypsination, lysed by sonication, and surviving bacteria in lysates determined by plating the lysates on TSA plates supplemented with sheep blood (TSA-SB) and counting the colonies that have grown on the following day. **a:** Representative images of TSA-SB plates after 24 h of incubation at 37°C. Scale bar, 5 mm. Arrows indicate colonies with SCV phenotype (≤10-fold smaller colony areas than those of the wild-type phenotypes, respectively). **b:** The percentage of colonies with SCV phenotypes formed on TSA-SB plates (between 150 and 400 colonies examined in each sample). Data are presented as box and whisker plot (n=5-6 biological replicates). ns, not significant (Mann-Whitney *U* test).
